# Complement, Coagulation, and Fibrinolysis: The Role of the Endothelium and Its Glycocalyx Layer in Xenotransplantation

**DOI:** 10.3389/ti.2024.13473

**Published:** 2024-10-15

**Authors:** Mitra Gultom, Robert Rieben

**Affiliations:** Department for Biomedical Research, University of Bern, Bern, Switzerland

**Keywords:** xenotransplantation, endothelium, endothelial glycocalyx, complement and coagulation, plasma cascades

## Abstract

In xenotransplantation, the vascular endothelium serves as the first point of contact between the recipient’s blood and the transplanted donor organ. The loss of the endothelium’s ability to control the plasma cascades plays a critical role in the dysregulation of the complement and coagulation systems, which greatly contribute to graft rejection and hinder long-term xenograft survival. Although it is known that an intact glycocalyx is a key feature of a resting endothelium that exhibits optimal anticoagulant and anti-inflammatory properties, the role of the endothelial glycocalyx in xenotransplantation is barely investigated so far. Here, we discuss the central role of endothelial cells and the sugar-rich endothelial glycocalyx in regulating the plasma cascades, and how the loss of these functions contributes to graft damage and rejection. We highlight the importance of preserving the regulatory functions of both endothelial cells and the glycocalyx as strategies to improve xenotransplantation outcomes.

## Introduction

The luminal side of the vascular endothelium is lined by a monolayer of endothelial cells (ECs), which function far beyond merely acting as the barrier between blood and tissue. EC functions include regulating the vascular tone, mechanotransduction, permeability, oxygen and nutrient supply, vascular hemostasis, as well as immunomodulatory activities [1–6]. The outermost layer of ECs is covered by the endothelial glycocalyx, a sugar-rich layer composed of diverse sugar conjugates, such as glycoproteins, proteoglycans, and glycolipids, which serve as the first point of contact for cellular and humoral components of the immune system with the endothelium [7–10].

The well-balanced control of the plasma cascade systems – complement, coagulation, fibrinolysis, and kallikrein/kinin – by ECs is crucial in healthy and pathological conditions, including pig-to-human xenotransplantation [11]. However, this control is lost due to incompatibilities between regulatory factors provided by the porcine ECs and the human plasma components present in the recipient’s blood, ultimately leading to graft damage and rejection [12–14]. The activation of the xenograft ECs and subsequent loss of the regulatory control of the plasma cascades have been shown to be the significant events leading to organ failure and rejection [15–19].

Genetic modifications have been used to overcome xenorejection due to humoral responses by eliminating the synthesis of xenoantigens [20–22]. In addition, the expression of human complement and coagulation regulatory genes in donor pigs has been used to regain control over the activation of the plasma cascade systems [23–26]. While impressive progress has been achieved, complement and coagulation dysregulations are still presented as key features of short- or long-term xenograft rejection in preclinical and clinical models [26–30]. This indicates that the currently available strategies to preserve the regulatory functions of xenograft ECs are not sufficient to achieve long-lasting control and graft survival.

In this review we outline the critical roles of ECs and the glycocalyx in regulating the plasma cascade systems. We also highlight the interaction of the EC glycocalyx with the recipient’s plasma cascades as well as its role in xenograft rejection. Finally, we discuss the potential of EC glycocalyx protection as a possible therapeutic strategy to improve graft survival and function.

## Regulation of Plasma Cascades by Endothelial Cells

Normal ECs are typically in a quiescent state, displaying minimal proliferation, migration, and permeability. The surface of healthy ECs is also in an anticoagulant, anti-inflammatory, and pro-fibrinolytic state. The maintenance of this quiescent state is achieved by complex and active functions involving the continuous production and regulation of various molecules and receptors [1, 11].

### Coagulation and Fibrinolysis

Coagulation and fibrinolysis are essential physiological processes that occur continuously, maintaining the delicate equilibrium between fibrin formation and breakdown in the plasma [31]. Activation of the coagulation cascade can be initiated via two pathways: the intrinsic pathway, also called the contact activation pathway, and the extrinsic pathway, which is triggered by tissue factor (TF). Both pathways result in the activation of factor X and subsequent production of thrombin, a serine protease that converts fibrinogen into fibrin, ultimately leading to clot formation. The extrinsic pathway mainly serves as a mechanism for hemostatic control and response to injury, while the activation of the intrinsic pathway has been associated with pathological clotting [32, 33].

Under normal physiological conditions, ECs prevent thrombus formation by providing a non-adhesive surface to prevent the activation of platelets and the coagulation cascade. More importantly, ECs are actively preventing thrombus formation by expressing soluble and membrane-bound molecules with anticoagulant properties [34]. ECs produce soluble molecules such as nitric oxide (NO) and prostacyclin (PGI_2_) which inhibit platelet aggregation [35–37]. To inhibit coagulation via the TF pathway, ECs also express tissue factor pathway inhibitor (TFPI) [38–40]. Furthermore, as the formation of fibrin happens continuously in the plasma, ECs prevent the accumulation of fibrin by secreting tissue-type plasminogen activator (tPA) and urokinase-type plasminogen activator (u-PA) [41, 42]. These two molecules will convert plasminogen to the active plasmin, which breaks down fibrin clots accumulating on the EC surface. To further enhance its anticoagulant properties, ECs bind inhibitors of coagulation factors, preventing the progression of the coagulation cascade. For instance, heparan sulfate (HS), present on the EC glycocalyx, bind the liver-derived antithrombin III (ATIII), which inhibits coagulation by preventing thrombin and other coagulation factors from binding to their substrates [43–45]. Moreover, the membrane-bound thrombomodulin (TBM) expressed on the EC surface can bind circulating thrombin and inhibit its procoagulant function, altering thrombin’s affinity from binding fibrinogen to binding and activating anticoagulant protein C [34, 46, 47]. ECs also express a high affinity receptor for protein C (EPCR), which binds protein C and further enhances its TBM-thrombin complex-mediated activation [48]. The coagulation inhibitory properties of ECs are summarized in (Table 1).

**TABLE 1:** Anticoagulant properties of endothelial cells.

Molecule	Form	Target action	Function	Ref
Nitric Oxide (NO)	Soluble gas	Platelets	Diffuses to nearby platelets and prevents calcium release, which ultimately prevents platelet activation and aggregation	[36, 37]
Prostacyclin (PGI_2_)	Soluble protein	Platelets	Inhibition of platelet activation through calcium release inhibition	[35]
Heparane Sulfate (HS)	Membrane-bound glycosami noglycans	ATIII	Bound ATIII inhibits thrombin (factor II) and other serine proteases binding to their substrates	[43, 44]
Tissue Factor Pathway Inhibitor (TFPI)	Soluble protein	Tissue factor (Factor III)	Limits the TF (factor III) activity which initiates prothrombin transformation into thrombin	[38, 39]
Thrombo modulin (TBM)	Membrane-bound protein	Thrombin, protein C	Binds to thrombin and reduces the ability of thrombin to convert fibrinogen into fibrin TBM-thrombin complex enhances protein C activity to inhibit coagulation factors Va and VIIIa	[46, 48]
t-PA	Soluble protein	Plasminogen	Activates plasminogen into plasmin and initiates fibrinolysis	[31, 41]
u-PA	Soluble protein	Plasminogen	Activates plasminogen into plasmin and initiates fibrinolysis	[31, 41]
EPCR	Membrane-bound protein	Protein C	Binds and promotes protein C activation by presenting protein C to TBM-thrombin complex, ultimately inhibiting factor Va and VIIIa	[48]

### Complement Cascade

The complement system plays a crucial role in clearing immune complexes and injured cells. It can be activated via three different activation pathways, all of which converge at the C3 level. Activation of the cascades produces opsonins (C3b, C4b), which mark the targets for subsequent removal, and the membrane attack complex (MAC, C5b-9), which directly lyses the target cells. Additionally, complement activation generates the anaphylatoxins C3a and C5a, promoting leukocyte recruitment and inflammation. However, the activation of the complement system acts as a double-edged sword because the effectors of complement activation have the potential to harm the host. To prevent this, the complement system is highly regulated through the expression of soluble and membrane-bound molecules to avoid the undesired effect of complement activation. ECs, as the first layer constantly exposed to the complement mediators in the plasma, play an indispensable role in this regulatory process.

ECs express membrane-bound components and secrete soluble molecules that prevent the activation of the complement cascade or deposition of complement activation products (Table 2). ECs express the surface molecules CD46 and CD55, which inhibit the activation of complement pathways, and CD59, which prevents the formation of MAC on the surface of ECs [50, 51, 54]. ECs also secrete C1 inhibitor (C1-INH), clusterin, factor H, and factor I, which prevent the formation of complement effectors at various stages [49, 52, 53, 55, 56].

**TABLE 2:** Complement regulatory properties of endothelial cells.

Molecule	Form	Target action	Function	Ref
C1 inhibitor	Soluble protein	C1	Inhibits the activation of complement C1	[49]
CD46 (MCP)	Membrane-bound protein	C3, C4	Mediates the cleavage of C3b and C4b	[50]
CD55 (DAF)	Membrane-bound protein	C3 convertase C5 convertase	Destabilizes C3/C5 convertases in the classical and the alternative pathways	[51]
Factor H	Soluble protein	C3b	Inactivates the C3 and C5 convertases	[52]
Factor I	Soluble protein	C3b, C4b	Cofactor for the C3b and C4b cleavage by factor H	[53]
CD59	Membrane-bound protein	C5b-9	Prevents C5b-9 formation	[54]
Clusterin	Soluble protein	C5b-9	Prevents C5b-9 formation	[55, 56]

Both the complement and coagulation systems comprise of serine proteases with common ancestral genes [57, 58]. There is substantial evidence of crosstalk between complement and coagulation factors, leading to mutual engagement of both systems. For instance, proteases such as thrombin, factor XII (Hageman factor), and plasmin play roles in both coagulation and complement activation [59–62]. This crosstalk also influences the regulation of the plasma cascade pathways by ECs through shared complement-coagulation regulators such as C1-INH, TBM, TFPI, CD46, CD55, and CD59 [58, 63–65]. Consequently, a lack of function of the previously mentioned inhibitors, can also contribute to concurrent activation and amplification of complement and coagulation pathways under pathological conditions, resulting in excessive activation of both systems [64, 66, 67].

### Genetic Modification Strategies to Overcome Complement and Coagulation Dysregulation in Xenotransplantation

In xenotransplantation, interaction of the porcine ECs with the human plasma cascades presents great immunological challenges due to the presence of xenoantigens on porcine ECs, and molecular incompatibilities between porcine plasma cascade regulators and human plasma cascade components [21, 22, 68]. The porcine glycocalyx presents sugars carrying α-Gal, N-glycolylneuraminic acid (Neu5Gc), and Sd^a^ epitopes, which can be recognized as foreign by natural antibodies circulating in human blood, and have been shown to play a significant role in the immune response to pig grafts in xenotransplantation [22, 68–71]. Antibody-mediated complement activation will lead to hyperacute rejection, a rapid destruction of the xenograft due to the complement attack, accompanied by interstitial hemorrhage, edema, and microvascular thrombosis [21, 72, 73]. The lack of complement and coagulation protection on the surface of ECs due to the interspecies molecular incompatibilities also leads to complement-mediated injury and coagulopathy, which are evident in various stages of xenograft rejection [74–76].

**FIGURE 1:**
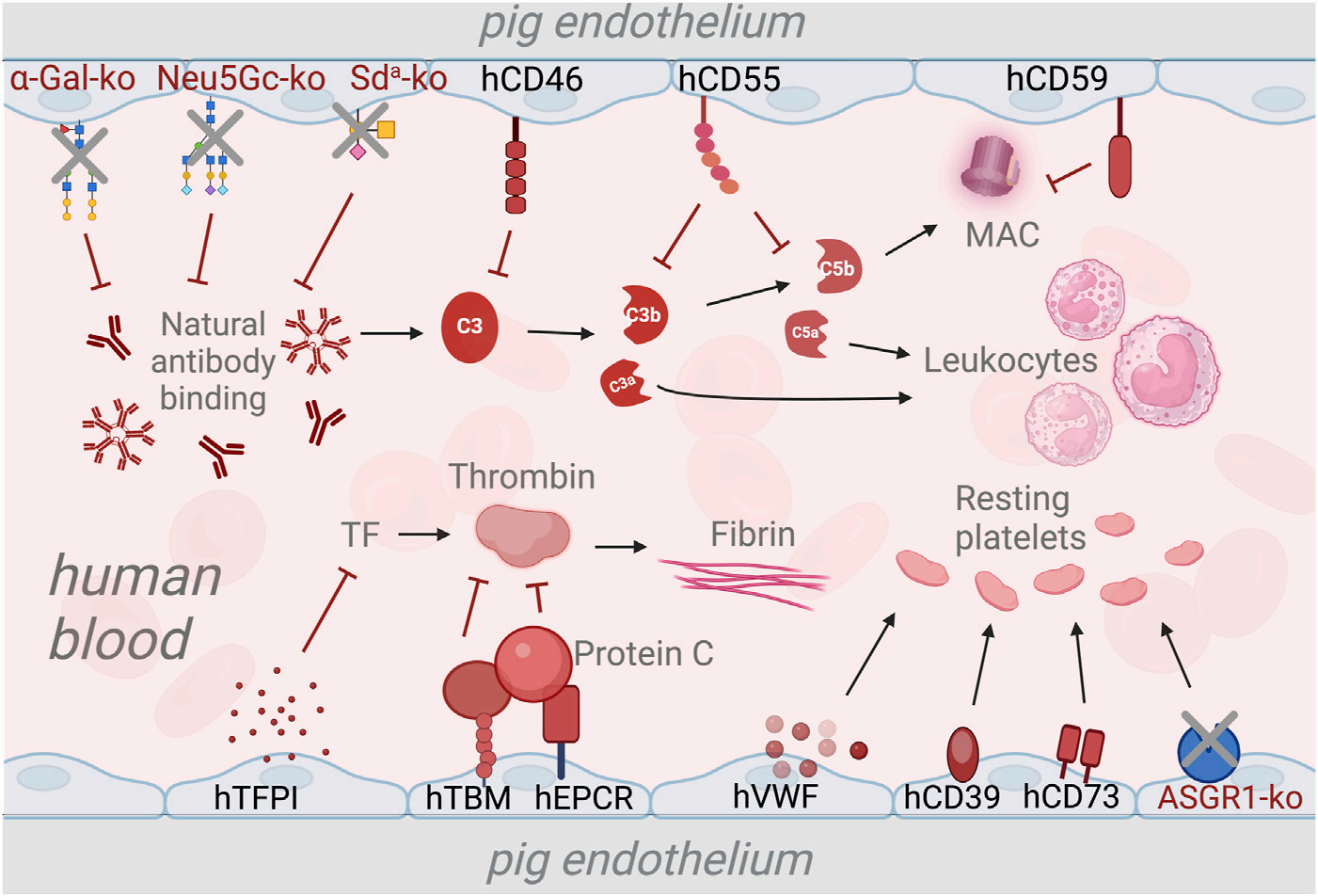
Strategies to overcome the complement and coagulation dysregulation in xenotransplantation using genetic modifications. The deletion of genes responsible for xenoantigen epitopes prevents the binding of natural antibodies and subsequent activation of the complement system [20, 21, 24]. Transgenic expression of human complement regulatory genes such as human CD46 (hCD46), hCD55, and hCD59 inhibits the activation of the complement cascade and the formation of membrane attack complex (MAC) [75, 78, 79]. The expression of human tissue pathway inhibitor (hTFPI) is used to prevent activation of the coagulation cascade via the tissue factor (TF) pathway [80]. Additionally, Human thrombomodulin (hTBM) and endothelial protein C-receptor (hEPCR) are expressed to inhibit thrombin activation, thus preventing fibrin formation [26, 81]. To maintain the platelets in a resting state, transgenic expression of human von Willenbrand Factor (hVWF), triphosphate diphosphohydrolase-1 (human ENTPDase-1 or hCD39) and human CD73 [82–84]. Deletion of the asialoglycoprotein receptor-1 (ASGR1) gene is used as a strategy to reduce human platelet destruction by pig livers [85, 86].

The first approach to avoid activation of complement on the EC surface in xenotransplantation is the deletion of porcine xenoantigens, which was shown to prevent hyperacute rejection [20, 24, 70, 77]. Another approach to provide better control of the plasma cascades by the endothelium is the transgenic overexpression of human complement and coagulation regulatory factors [16, 23–26]. Strategies to overcome the complement and coagulation dysregulation are illustrated in Figure 1. Genetically modified pigs carrying single or multiple xenoantigens knockouts and expressing varying combination of plasma cascade regulatory proteins have been developed and show different survival rates [26, 81, 87–89].

## The Role of the Endothelial Glycocalyx in Plasma Cascade Regulation

The function of ECs, from mechanotransduction, maintenance of vascular integrity and vascular tone, as well as regulating the interaction of EC with the immune components of the blood, is profoundly influenced by their glycocalyx component [90]. While the term glycocalyx broadly refers to surface sugar layer of any cells, the EC glycocalyx specifically refers to the carbohydrate-rich layer on the luminal side of ECs. The thickness and the diversity of the EC glycocalyx is influenced by the shear stress, plasma proteins, and extrinsic glycosylation factors in the blood [3, 10, 91–93]. The EC glycocalyx is made up of diverse sugar conjugates, including proteoglycans, glycoproteins, and glycolipids, facilitating the interaction of ECs with various immune mediators of the plasma cascades.

Proteoglycans are glycoproteins comprising a core protein (e.g., syndecan, glypican, and perlecan) that carries covalently attached linear polysaccharide glycosaminoglycans (GAGs). The main components of the EC glycocalyx GAGs are HS, chondroitin sulfate (CS) and dermatan sulfate (DS) [10, 94]. The EC glycocalyx also contains the non-sulfated GAG hyaluronan (HA), typically anchored by the CD44 receptor, or as a soluble component [95, 96]. GAGs are the most abundant sugars in the EC glycocalyx, contributing significantly to its molecular mass and negative charge, creating a transendothelial gradient for fluid transit, masking the adhesion molecules, and capturing circulating plasma proteins [97, 98]. GAGs are also essential in maintaining the quiescent state of ECs. They act as mechanotransducers, signaling to maintain EC function via the production of NO [90, 99]. GAGs can bind and modulate the activity of several growth factors, antiangiogenic, and inflammatory molecules such as VEGF, FGF, IL8, MMPs, angiopoietin-1, thrombospondin-1, endostatin, and PAI-1 [100–105]. Additionally, GAGs have binding sites for xantine-oxydoreductase (XOD) and endothelial superoxide dismutase (eSOD), preventing oxidative damage to ECs [106, 107].

HS is one of the most extensively studied GAGs and builds up most of the sugar components of the EC glycocalyx. HS is known for its critical role in various biological functions, from serving as a binding domain for signal transduction molecules to regulating the plasma cascade and immune interactions [108]. Plasma proteins bind to HS via their heparin-binding domain. This binding is specific, and through the differential expression of HS, ECs can selectively determine which plasma proteins will bind to the surface [109, 110]. HS on the EC glycocalyx can bind and potentiate the activity of complement and coagulation regulatory factors, including factor H, C1-INH, and ATIII, providing an anti-inflammatory and anticoagulant surface [44, 111–115]. Binding of growth factors and cytokines such as VEGF, FGF, and IL8 is predominantly facilitated by HS [43, 103, 116, 117]. HS also serves as a binding site for P-selectin and L-selectin, potentially acting as a decoy receptor to influence cell adhesion dynamics [118, 119]. Moreover, HS is also involved in the regulation of the kinin-kallikrein system through its capacity to recruit kinin precursors and mediate the activation of high molecular weight kininogen [120, 121].

The terminal glycans of mammalian glycoproteins and glycolipids are typically substituted by the negatively charged sialic acid, which plays an essential role in physiological and pathological processes, including complement, coagulation, and inflammation [72, 122–124]. Many important receptors and regulatory proteins on the EC surface carry N- and O-linked oligosaccharide chains and are classified as glycoproteins [94, 125]. The interaction between ECs and leukocytes is controlled by adhesion molecules such as siglecs and selectin receptors, that are heavily influenced by the cellular glycosylation patterns of these proteins [126, 127]. Factor H can bind to sialic acid, providing a protective layer on ECs and shielding the cells from the deposition of complement [128–131]. Glycosphingolipids (GSLs), a diverse group of glycolipids containing one or more glycans anchored to a ceramide, are known to play important roles in many cellular processes, such as signal transduction, cell-cell interactions, and immune response regulation, including the complement and coagulation systems [132–135]. While the role of GSLs has been extensively studied in neurodegenerative diseases, their role in the context of xenotransplantation immunology is less well-understood.

### Glycocalyx Shedding

Due to its pivotal role in maintaining the normal function of ECs, destruction of the EC glycocalyx will lead to impairment of EC function and overall vascular hemostasis. Glycocalyx shedding is observed when ECs are activated and exhibit a pro-inflammatory state, including in trauma-related injury, ischemia/reperfusion injury, hemorrhagic shock, and hyperglycemia [10, 136–139]. In xenotransplantation, glycocalyx shedding associated with complement and coagulation activation after xenogeneic treatment has been observed *in vitro* and *in vivo* [140–144]. Disruption of the EC glycocalyx may hamper its mechanotransduction ability and induce edema due to elevated vascular permeability [145–149]. Shedding of the EC glycocalyx will result in the loss of anticoagulant and anti-inflammatory properties of the EC, leading to increased coagulation, decreased pro-fibrinolytic activity, increased complement deposition, and adhesion of leukocytes to the EC surface [138–140, 150].

Shedding of the EC glycocalyx during inflammation can be mediated by various glycocalyx modifying factors such as reactive oxygen species (ROS), matrix metalloproteinases (MMPs), and glycan-degrading enzymes (heparanase, hyaluronase, sulfatase, sialidase, etc.) [9, 10, 139, 151]. Other proteins with enzymatic functions such as thrombin, elastase, proteinase 3, plasminogen, as well as cathepsin B also contribute to degradation of the glycocalyx [152–154]. In cardiovascular diseases associated with vascular inflammation, the observed glycocalyx shedding can be mediated by multiple glycocalyx-degrading mediators [139, 155, 156].

### Interaction of the Porcine Glycocalyx With Human Plasma Proteins in Xenotransplantation

**FIGURE 2:**
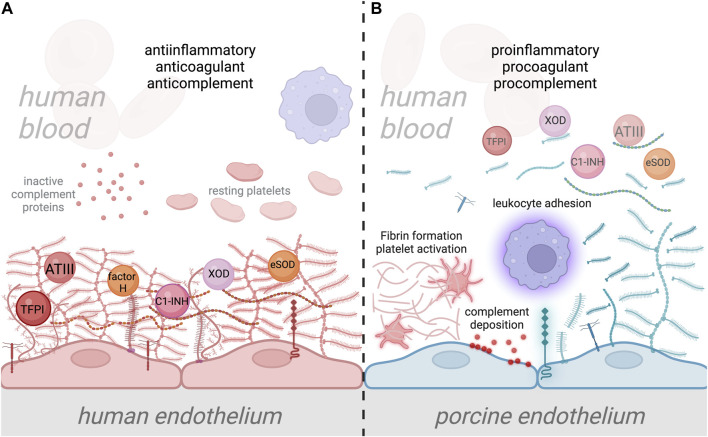
Interaction of human plasma cascade proteins with human and porcine glycocalyx. **(A)** Normal interaction between human plasma proteins and the human endothelial cell (EC) glycocalyx, where plasma cascade regulatory molecules such as antithrombin III (ATIII), tissue factor pathway inhibitor (TFPI), Factor H, and C1-inhibitor (C1-INH) are specifically bound to EC glycocalyx, providing anticomplement, anticoagulant, and anti-inflammatory properties to the ECs [44, 112–114]. The human EC glycocalyx also binds antioxidant molecules such as XOD and SOD, thereby preventing oxidative damage to ECs [106, 107]. Attachment of leukocytes is prevented by the negatively charged EC glycocalyx and the shielding of leukocyte binding receptors [138, 157]. **(B)** In xenotransplantation, the disparity between human and porcine EC glycocalyx composition, which likely alters its structure and function, could lead to inefficient binding of protective plasma proteins. This could result in the transition of the endothelium from a quiescent into an activated state, which leads to glycocalyx shedding. Disruption of the porcine EC glycocalyx and loss of its anticoagulant properties will result in activation of the coagulation system, platelet recruitment and fibrin formation. Loss of anticomplement properties will also lead to the deposition of complement proteins on the EC surface, along with the amplification of inflammation. Additionally, shedding of the porcine EC glycocalyx will increase leukocyte attachment to the surface of EC due to the loss of leukocyte binding receptor shielding. Ultimately, this condition could lead to xenograft damage and failure.

One significant concern, and barely studied so far, are the possible differences in the binding of human plasma proteins to the human and porcine glycocalyx (Figure 2). The absence of glycans containing *α*-Gal, Neu5Gc, and Sd^a^ epitopes in humans highlights the species-specific differences in sugar synthesis and expression, as well as the disparity in glycocalyx composition between humans and pigs [21]. Due to its different composition, the porcine glycocalyx may have varying structure and affinities for human plasma cascade regulators such as ATIII, factor H, and C1-INH, which could reduce the anti-inflammatory and anticoagulant properties of the xenografted EC surface once a porcine organ has been grafted into a human recipient. Discrepancies in the binding affinities to human growth factors, cytokines, and chemokines could likely also cause a systemic effect due to loss of control of dissociated and active molecules by ECs [103, 109, 158]. Similarly, possible differences in enzyme kinetics and affinities between the porcine glycocalyx and glycocalyx-modifying enzymes could also affect the glycocalyx dynamics and function. Therefore, despite the effort to substitute the interspecies complement and coagulation incompatibility on a protein level in xenotransplantation, EC function in controlling the plasma cascade regulation might still not be optimal without proper functioning of the glycocalyx [144].

Initially, the removal of sugar xenoantigens from xenograft donor pigs by genetic engineering was implemented to overcome the antibody-mediated rejection and has been proven effective [20, 24, 70, 77]. However, these genetic modifications have been found to influence the expression of other sugars and may even lead to the synthesis of novel glycans, which is likely to influence the structure, composition, and immunological function of the porcine EC glycocalyx [159, 160]. Therefore, further studies are necessary to determine how these alterations in sugar composition and the emergence of neoglycans influence the phenotype and physiological function of the porcine EC glycocalyx.

## Exploring EC Glycocalyx Protection in Xenotransplantation

Protecting ECs and their glycocalyx is critical for improving graft survival and function, highlighting the importance of targeted therapy for preserving and maintaining the quiescent state of the endothelium. Current strategies, including genetic engineering, might indirectly protect the glycocalyx to a certain extent by preventing antibody binding, complement activation, and controlling the coagulation system. Additionally, anti-inflammatory and anticoagulant agents, such as corticosteroids and heparin, which are already incorporated in current immunosuppressive regimens in xenotransplantation, have been shown to prevent the degradation of the glycocalyx [161–163]. However, although evidence specific to xenotransplantation remains limited, shedding of the glycocalyx continues to be a significant issue in pathological conditions related to vascular inflammation. Shedding of the glycocalyx in a setting of (xeno)transplantation occurs immediately after graft reperfusion (ischemia reperfusion injury), related to trauma and inflammation, or long-term related to chronic rejection [136–138, 156, 164, 165]. Therefore, strategies to prevent shedding of the glycocalyx, or to functionally replace glycocalyx components, might be needed in clinical xenotransplantation.

Generally, glycocalyx protection strategies employ therapeutic agents that interact with one or multiple glycocalyx-modifying factors, including inhibiting the glycocalyx-degrading enzymes, oxidative stress, and inflammation. Molecules such as angiopoietin, hydrocortisone, ATIII, berberine, and S1P prevent the degradation of glycocalyx through the inhibition of MMPs [10, 153, 166, 167]. SOD acts as antioxidant and helps to mitigate oxidative stress associated with ischemia-reperfusion injury, thus protecting the glycocalyx [149]. Sulodexide can protect the glycocalyx by inhibiting heparanase-1 [168]. While those therapies have been used in other vascular disorders, the protective effects on porcine xenografts need further evaluation, especially since existing studies do not fully address the structure, function, and dynamics of the glycocalyx in a xenotransplantation context.

Glycan-specific approaches have also been employed for glycocalyx protection and restoration. Emerging therapies such as bioengineering approaches also offer innovative strategies, for instance, by engineering the EC to rebuild the glycocalyx, which has been shown to minimize graft injury and rejection [169]. Other strategies use a class of molecules which can be termed “endothelial cell protectants.” One such molecule, low molecular weight dextran sulfate of 5000 MW (DXS), was shown to be protective both *in vitro* and in a small animal model of xenotransplantation [170, 171]. DXS has also been shown to prevent ischemia/reperfusion injury in large animal models [172, 173]. Similarly, multimeric sulfated tyrosine has been shown to help maintain and restore the glycocalyx layer [174]. While currently there are no clinically approved drugs which act as EC protectants, this class of substances has proved to be successful in preclinical experiments and, in our view, by keeping the endothelium in a quiescent state, has great potential as an additional drug treatment in xenotransplantation.

## Conclusion

The interaction between the porcine glycocalyx and the human plasma cascades involves complex, yet poorly understood dynamics that are critical to the success of xenotransplantation. To address these challenges, a deeper understanding of interspecies glycocalyx differences and their impact on immune responses, coagulation, and EC function is required. New insights into the structure and function of the porcine and human glycocalyx, as well as the mechanisms of glycocalyx degradation and regeneration in xenotransplantation, hold the potential to unlock novel approaches to preserve the endothelial glycocalyx. Protective strategies for the glycocalyx, combined with the already available strategies to prevent complement and coagulation dysregulation, can be explored as an approach to improving the outcomes of xenotransplantation.

## Biography

Robert Rieben was born in Bern, Switzerland, where he also studied biology. During his PhD he worked on blood group ABO antibodies and, via contacts with Rafael Oriol and David Cooper, got in touch with xenotransplantation. As a postdoc in Mohammed Daha’s lab in Leiden/NL, he started to work on the complement system and endothelial cells. At the same time, he collaborated in the first EU-funded xenotransplantation research projects “Glycoimmunology”, led by David Joziasse, and “Xenotransplantation”, led by Bo Samuelsson. Back in Switzerland, he started his own research group and continued to work on the concept of endothelial cell protection in xenotransplantation, allotransplantation, and ischemia/reperfusion injury. For the past 12 years, Robert Rieben has been affiliated to the German Transregio 127 research project on xenotransplantation, led by Bruno Reichart and Eckhard Wolf, and since 2020 he leads a Sinergia project of the Swiss National Science Foundation together with Joerg Seebach and Eckhard Wolf.
